# Genome-Wide Characterization of DNA Methylation in an Invasive Lepidopteran Pest, the Cotton Bollworm *Helicoverpa armigera*

**DOI:** 10.1534/g3.117.1112

**Published:** 2018-01-02

**Authors:** Christopher M. Jones, Ka S. Lim, Jason W. Chapman, Chris Bass

**Affiliations:** * Vector Biology, Liverpool School of Tropical Medicine, L3 5QA, UK; † Computational and Analytical Sciences, Rothamsted Research, Harpenden, AL5 2JQ, UK; ‡ Biointeractions and Crop Protection, Rothamsted Research, Harpenden, AL5 2JQ, UK; § Centre for Ecology and Conservation, University of Exeter, Penryn, TR10 9EZ, UK; ** College of Life and Environmental Sciences, University of Exeter, Penryn, TR10 9EZ, UK

**Keywords:** cytosine methylation, epigenetics, phenotypic plasticity, flight activity, gene expression

## Abstract

The genes and genomes of insect pests are shaped by the wide array of selective forces encountered in their environments. While the molecular adaptations that evolve are beginning to be understood at the genomic and transcriptomic level, they have been less well characterized at an epigenetic level. Here, we present a genome-wide map of DNA methylation at single-nucleotide resolution for the cotton bollworm moth, *Helicoverpa armigera*, a globally invasive pest of agriculture. We show that methylation is almost identical in the larvae and adults of *H. armigera* and that, through whole-genome bisulfite sequencing (WGBS), at the most ∼0.9% of CpG sites in this species are methylated. We find that DNA methylation occurs primarily in exons, is positively correlated with gene expression, and that methylated genes are enriched for cellular “housekeeping” roles. *H. armigera* has an exceptional capacity for long-range migration. To explore the role of methylation in influencing the migratory phenotype of *H. armigera*, we performed targeted bisulfite sequencing on selected loci from 16 genes that were differentially expressed between adult moths exhibiting distinct flight performance in behavioral assays. While most CpG sites in these genes were not methylated between flight phenotypes, we identified hypermethylation in a demethylase (*KDM4*) that targets lysine-specific histone modifications, which are strongly associated with transcription and methylation. The *H. armigera* methylome provides new insights into the role of DNA methylation in a noctuid moth and is a valuable resource for further research into the epigenetic control of adaptive traits in this important pest.

DNA methylation is an ancient epigenetic modification that pervades a wide range of organisms. Despite the conserved biochemistry of methylation, its function and magnitude are highly variable across taxa with, for example, methylation levels three-orders of magnitude lower in the genomes of insects compared to those from the animal or plant kingdoms (Zemach *et al.* 2010). Furthermore, the catalog of DNA methyltransferases (DNMTs) found in animals—the enzymes needed to maintain (DNMT1) or catalyze *de novo* methylation (DNMT3)—differ between insect species and are completely absent in some cases [*e.g.*, *Drosophila melanogaster* (Raddatz *et al.* 2013)]. Nevertheless, the presence of a functional DNA methylation system across the class Insecta with conserved patterns of methylation (Sarda *et al.* 2012; Hunt *et al.* 2013a; Bewick *et al.* 2016) suggests an important, although poorly understood, role for this epigenetic mark on the biology of insects.

Advances in whole-genome sequencing coupled with bisulfite DNA treatment have led to single-nucleotide resolution maps of methylation in a range of invertebrates (Lyko *et al.* 2010; Xiang *et al.* 2010; Wurm *et al.* 2011; Wang *et al.* 2013, 2014). These studies have shown that insect methylation is primarily confined to CpG dinucleotides (cytosine followed by guanine), occurs primarily in gene bodies (exons + introns), and that hypermethylated genes are generally associated with cellular housekeeping roles, whereas hypomethylated genes are more tissue specific (Sarda *et al.* 2012). Experimental measurements of methylation mirror those inferred indirectly from the computation of the observed to expected CpG ratio (CpG O/E) which measures the depletion of CpG dinucleotides (Bird 1980). The CpG O/E is lower in methylated genes due to the mutagenic conversion of methylated cytosine to thymine (deamination) over time, leaving a historical imprint of methylation. A bimodal distribution of CpG O/E has been shown in several insects, indicating the presence of two classes of “lowly” and “highly” methylated genes, and has been used as evidence for active genome-wide methylation.

There is a clear positive correlation between robust intragenic methylation and constitutive gene expression in insects (Xiang *et al.* 2010; Hunt *et al.* 2013b; Libbrecht *et al.* 2016). This relationship is strengthened in the context of nucleosome dynamics with spatial concordance between methylation and an additional epigenetic marker, histone post-translational modifications, which are thought to act in concert to regulate transcriptional activity (Hunt *et al.* 2013a; Glastad *et al.* 2015). Intragenic DNA methylation is therefore thought to regulate active transcription in insects, but whether this is restricted to conserved genetic pathways or can be extended to influence a phenotypic response is still relatively unknown.

Phenotypic plasticity in morphological and behavioral traits represents a promising role for DNA methylation in insects, yet, evidence for this remains equivocal. In eusocial insects, such as honeybees and ants, evidence that methylation drives caste differentiation (*e.g.*, development into a worker or queen bee) has been provided through whole-genome sequencing and *DNMT* silencing (Kucharski *et al.* 2008; Lyko *et al.* 2010), but this has recently been challenged (Wang *et al.* 2013; Libbrecht *et al.* 2016; Standage *et al.* 2016). Other promising examples of behaviors that may be impacted by methylation are those exhibited in response to shifting or deteriorating environments, such as dispersal or migration. A comparative methylome analysis of the brain tissue from the solitarious and density-dependent gregarious forms of the migratory locust (*Locusta migratoria*) showed that differentially methylated genes were associated mainly with synaptic plasticity (Wang *et al.* 2014). Furthermore, genes differentially expressed between the gregarious and solitarious phases were shown to have signs of CpG depletion (Robinson *et al.* 2011), and genes encoding methylation machinery (*DNMT1*, *DNMT2*, and *methyl CpG binding domain protein 2/3*) are differentially transcribed in certain tissues of the two phases (Robinson *et al.* 2016). Finally, beyond insects, differentially methylated regions have been identified between migratory and nonmigratory life stages of other organisms, such as the rainbow trout *Oncorhynchus mykiss* (Baerwald *et al.* 2016), suggesting that the development of migratory forms in response to environmental cues may be linked to variation in methylation patterns.

The Old World bollworm (*Helicoverpa armigera*), is a globally distributed agricultural pest noctuid moth that causes considerable economic damage worldwide (Kriticos *et al.* 2015). More recently, *H. armigera* has invaded the “New World,” with evidence of multiple incursions occurring in South America, and subsequent spread over the continent and into Central America (Tay *et al.* 2017). The invasiveness of *H. armigera* is accentuated by adaptive life-history strategies, such as extensive polyphagy (Cunningham and Zalucki 2014), resistance to insecticides and Bt toxins (Downes *et al.* 2016), and facultative long-range migratory movements (Farrow and Daly 1987). The recent release of the *H. armigera* genome has shown that gene loss and transcriptional plasticity have facilitated polyphagy in this species (Celorio-Mancera *et al.* 2012; Pearce *et al.* 2017), and similar processes may underlie other traits, including long-distance migration (Jones *et al.* 2015). However, the role of epigenetic processes in regulating the life history of important Lepidoptera is virtually unknown. Common to the Lepidoptera, *H. armigera* possesses DNMT1 but lacks the *de novo* methylase DNMT3, although it is becoming clear that the association between the presence of specific DNMTs and methylation is not binary, and indeed, DNMT1 may compensate for the lack of DNMT3 in some cases (Bewick *et al.* 2016). A map of methylation levels in this species would complement insights from the recently published genome (Pearce *et al.* 2017).

Here, we present a detailed analysis of the methylome of *H. armigera* through WGBS and analyze the patterns of methylation in the context of published insect methylomes to date. Previously, we have shown that the flight propensity of *H. armigera* is associated with the differential expression of a suite of candidate genes associated with lipid metabolism, flight muscle function, and hormonal control (Jones *et al.* 2015). Therefore, we extended our analyses using targeted bisulfite sequencing to investigate potential methylation differences in a subset of these genes between insects demonstrating distinct flight performances in behavioral assays.

## Materials and Methods

### Mass spectrometry total DNA methylation analysis

Selected reaction monitoring mass spectrometry (SRM MS) was used to quantify global levels of 5-hydroxymethyl-2’-deoxycytidine (5HmdC) and 5-methyl-2’-deoxycytidine (5mdC). The assay measures 5HmdC and 5mdC concentrations as a percentage of 2’-deoxyguanosine (dG). The calibrated ranges for the analytes were 0–1.25% for 5HmdC and 0–12.5% for 5mdC using a fixed 40 pmol amount of dG as an internal standard. MS was performed on genomic DNA (gDNA) extracted from either the heads and thoraxes of four adult moths (two males and two females) or four larvae (L3 life stage). gDNA was extracted using the EZNA Insect DNA Kit (Omega Biotek) and treated with RNaseA (Thermo Life Sciences). Three biological replicates (pools of four insects) per life stage were analyzed.

### WGBS

For the methylome analysis, gDNA was extracted from the heads and thoraxes of four male and four female *H. armigera*, and pooled for sequencing. Insects were collected as adults from Bt cotton fields in Qiuxian (Hebei province, China, 36.81°N, 115.16°E) and reared for one generation in the insectaries at Rothamsted Research. Adults were snap-frozen in liquid nitrogen and gDNA extracted using the DNeasy Blood and Tissue Kit (QIAGEN). Methyl-MaxiSeq (Zymo Research) libraries were prepared from 100 ng of bisulfite-treated gDNA (EZ DNA Methylation-Lightning Kit). Bisulfite-converted DNA was amplified with a primer that contained part of an adaptor sequence plus four random nucleotides, followed by two additional amplifications to add on the remaining adaptor sequence and to barcode the fragments. PCR products were purified using the DNA Clean & Concentrator-5 (Zymo Research). Sequencing was run on the Illumina HiSequation 2500 platform. Sequence reads were aligned to the *H. armigera* genome using the bisulfite sequencing aligner software Bismark (Krueger and Andrews 2011).

### CpG methylation and gene methylation analysis

The methylation status of each cytosine was determined using a binomial distribution to compare methylated and nonmethylated reads at each site possessing a minimum of two reads (Lyko *et al.* 2010). Methylated sites were determined at *P* < 0.05 after adjustment for multiple testing (Benjamini and Hochberg 1995). Methylation ratios (mCpG/CpG) were determined per gene and genomic function (exon, intron, and 2 kb upstream). Methylated genes or genomic functions were defined as those possessing a methylation ratio of > 10%. CpG depletion (CpG O/E) was calculated according to Bird (1980). Hartigan’s diptest was used to determine the modality of the distribution of methylation levels and CpG O/E using the diptest package in R software (Hartigan and Hartigan 1985). An enrichment analysis (Fisher’s exact test) of GO terms for highly methylated genes (> 50% methylation ratio) and genes containing zero methylated sites was performed against the reference gene set in Blast2GO at an FDR < 0.05 (Gotz *et al.* 2008).

The relationship between methylation and gene expression was explored using the RNA-seq data set from Jones *et al.* (2015). Genome-wide expression data were acquired from a population collected in Anyang (Henan province, 36.10°N, 114.20°E). Anyang is ∼100 km from Qiuxian and insects were collected at a similar time of year (August 2013); therefore, it is expected that population differences between the two groups of insects were minimal. The relationship between methylation and expression was explored using the methylation ratio and TMM-normalized FPKM values (fragments per kilobase of exon per million fragments mapped).

### Flight mills and targeted bisulfite sequencing

To validate the genome-wide bisulfite data, and to determine the strength of any association between DNA methylation and flight activity, targeted bisulfite sequencing was performed on adult moths flown on tethered flight mills. Female moths originating from northern Greece were flown on the tethered flight mills following the procedures outlined in Jones *et al.* (2015). Insects were flown overnight and flight data collected between the hours of 1900 and 0915 (dark period 2000–0600). Individuals were snap-frozen in liquid nitrogen 1–2 hr following the flight period for DNA extraction. Following an analysis of the flight behavior, a total of 16 individuals (all female) representing two distinct groups of short- and long-distance fliers (eight in each phenotype) were chosen for DNA extraction and targeted bisulfite sequencing.

A selection of loci spanning 16 candidate genes, capturing a range of methylation (exon methylation ratio 0.025–1), were chosen for the detection of CpG sites. Primers were designed with parameters that preferentially targeted regions between 100 and 300 bp and avoided annealing to CpGs. Details of selected gene regions and primer design are available in Supplemental Material, Table S1. gDNA from 16 individual moths was extracted from the head and thorax using the EZNA Insect DNA Kit (Omega Biotek) as described above. Samples were bisulfite converted using the EZ DNA Methylation-Lightning kit (Zymo Research) and purified (ZR-96 DNA Clean & Concentrator, Zymo Research). Bisulfite-treated DNA (5 ng) was amplified, and the amplicons pooled for barcoding and sequencing using a MiSeq V2 300 bp Reagent Kit (Illumina).

Low-quality reads and adapter sequences were trimmed, and the sequencing reads realigned to the *H. armigera* genome using Bismark (Krueger and Andrews 2011). Nucleotides in primers were trimmed in the methylation calling and the methylation level quantified as the number of reads reporting a cytosine divided by the total number of reads at that site. Only CpG sites detected in at least one sample with at least 10 reads were considered for analysis. The fractional methylation ratio was calculated as the number of methylated cytosines (mCpG) over number of cytosines per site (mCpG/CpG). Mean differences between the two groups of individual moths displaying contrasting performances on the flight mills (*N* = 8) were estimated using a Student’s *t*-test.

### Data availability

The raw bisulfite sequencing data used to analyze the methylome is available at ArrayExpress (accession number E-MTAB-4779). Table S1 provides information on the primers used to amplify selected loci for targeted bisulfite sequencing. Table S2 describes the enriched GO terms in highly methylated genes and Table S3 lists enriched GO terms in genes with no detectable methylation. Table S4 shows the top 25 differentially expressed genes associated with flight activity per methylation level. Table S5 details the selected loci for targeted bisulfite sequencing. Table S6 provides all the raw CpG data per individual site and single gene, and the total exonic and intronic methylation ratio per gene. File S1 contains Figures S1–S4.

## Results and Discussion

### MS detection of global CpG methylation levels in H. armigera

The total levels of 5mdC and H5mdC were measured using MS in adults and larvae. Despite missing DNMT3, methylation is observed in *H. armigera* and the percentage of 5mdC was almost identical in the two life stages (adults, 0.165% ± 0.009 and L3 stage larvae, 0.164% ± 0.009), whereas H5mdC was undetectable in *H. armigera*. This suggests that DNA methylation is stable across life stages of *H. armigera* and, in contrast to recent findings in the honeybee (Wojciechowski *et al.* 2014), there is no evidence for additional epigenetic regulation via hydroxymethylation in this species.

### WGBS of DNA methylation in H. armigera

Sequencing of bisulfite-converted gDNA from the heads and thoraxes of eight adult moths of *H. armigera* (four females and four males) yielded 529 million reads, of which 28% mapped to the genome. The overall bisulfite conversion rate was high (> 99%). Methylation in insects is almost exclusively at CpG dinucleotides rather than CHG or CHH sites (H = A, C, or T) (Lyko and Maleszka 2011), so we focused on methylation at CpG sites only. Of the estimated 19.7 million CpG sites in the *H. armigera* genome, 73.5% were identified by sequencing (*N* = 14.5 million) with an average coverage of 28×.

The number of mCpGs detected (probability of methylated cytosine according to a binomial distribution, *P* < 0.05, minimum two reads per site) was 169,911, which represents 0.86% of all CpGs in the genome and 1.17% of those identified from bisulfite sequencing. Using a stricter threshold of 10 reads per site, 0.43% of all cytosines detected and 0.34% of all cytosines in the genome were methylated. The distribution of mCpGs are presented for both thresholds in Figure S1 in File S1. Comparisons with other genome-wide bisulfite data require some caution due to differences in mCpG detection methodology; however, the absolute number of mCpGs detected in this study is similar to that predicted in *Bombyx mori* (169,911 *vs.* 172,117) (Xiang *et al.* 2010). However, based on the estimated number of genomic CpGs, the relative level of methylation is much greater in *H. armigera* (0.86% *vs.* 0.11%). Of the estimated 17,086 genes from the recently annotated *H. armigera* genome (Pearce *et al.* 2017), ∼69.6% have at least one mCpG site.

**Figure 1:**
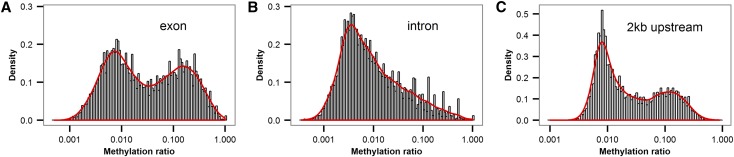
The distribution of the methylation ratios per genomic function. Distribution of methylation in (A) exons, (B) introns, and (C) 2 kb from the transcriptional start site.

Comparison of the level of CpG methylation in exons, introns, and the 2 kb region upstream of the gene transcription start site (TSS) (putative promoter region) (Figure S2 in File S1) revealed that exonic CpGs are more highly methylated (3.06%) than introns (0.57%) or the 2 kb upstream (1.78%), in line with previous findings that DNA methylation is primarily confined to exons in insects (Lyko *et al.* 2010; Xiang *et al.* 2010; Wang *et al.* 2013; Beeler *et al.* 2014; Bewick *et al.* 2016). The mean exon methylation ratio (calculated as the proportion of methylated cytosines determined by the binomial distribution) is also much higher than in introns and 2 kb upstream regions (exon mean, 0.053; intron mean, 0.017; and 2 kb upstream regions, 0.023). The distribution of the exon methylation ratio follows a bimodal distribution with two overlapping clusters of lowly and highly methylated genes, similar to the patterns of methylation reported for *B. mori* and *Apis mellifera* (Sarda *et al.* 2012) (Figure 1A). Intronic methylation ratios are, unimodal in line with patterns in other insects (Figure 1B). There is a small bimodal pattern in regions 2 kb upstream of the TSS, but this is probably insignificant given that methylation levels before the TSS are generally low in other Lepidopterans (Xiang *et al.* 2010) and could be due to inaccuracies in the annotation of intragenic regions of the genome (Figure 1C). The bimodal pattern of gene body methylation is a common feature between distantly related invertebrates with functional methylation systems (Sarda *et al.* 2012). These results also confirm that functional DNA methylation occurs in Lepidoptera despite the loss of DNMT3 from this order ∼177.99–116.45 MYA, and that either DNMT1 may compensate for this loss or *de novo* methylation occurs through some other non-DNMT-like protein (Bewick *et al.* 2016).

**Figure 2:**
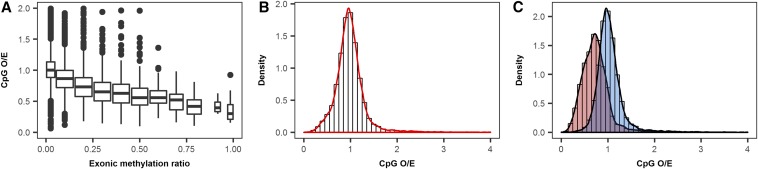
Patterns of methylation inferred from the CpG O/E (the observed to expected CpG ratio) statistic. (A) Correlation between the CpG O/E and experimentally deduced methylation ratio. (B) Unimodal distribution of CpG O/E. (C) Distribution of methylated (red) and nonmethylated (blue) genes (± 10% exon methylation ratio) per CpG O/E statistic.

There is negative correlation between exon methylation and the CpG O/E ratio (Figure 2A), reflecting the propensity for methylated cytosines to be converted to thymines over time (Bird 1980). In contrast to the bimodal distribution of exonic methylation in *H. armigera* described above and the CpG O/E ratio in other insects (Lyko *et al.* 2010; Walsh *et al.* 2010; Falckenhayn *et al.* 2013; Wang *et al.* 2014), we observe a single CpG O/E peak (Figure 2B; mean CpG O/E per gene 0.991). This is consistent with available CpG O/E distributions from other Lepidoptera (*B. mori* and *Danaus plexippus*) and the red flour beetle (*Tribolium castaneum*) (Xiang *et al.* 2010; Zhan *et al.* 2011). The common unimodel CpG O/E distribution in these species could be due to reduced CpG depletion over evolutionary time, potentially a result of the loss of the *de novo* methylation enzyme DNMT3 (Bewick *et al.* 2016). Nevertheless, when we classified genes as methylated or nonmethylated according to the level of exon methylation ratio (± 10%), there is a clear segregation into low CpG O/E (methylated) and high CpG O/E (nonmethylated) (Figure 2C), with significant differences between the mean CpG O/E of methylated (0.738) and nonmethylated genes (1.042) (*F*_2944,14055_ = 1.305, *P* < 0.0001).

### DNA methylation and gene expression in H. armigera

**Figure 3:**
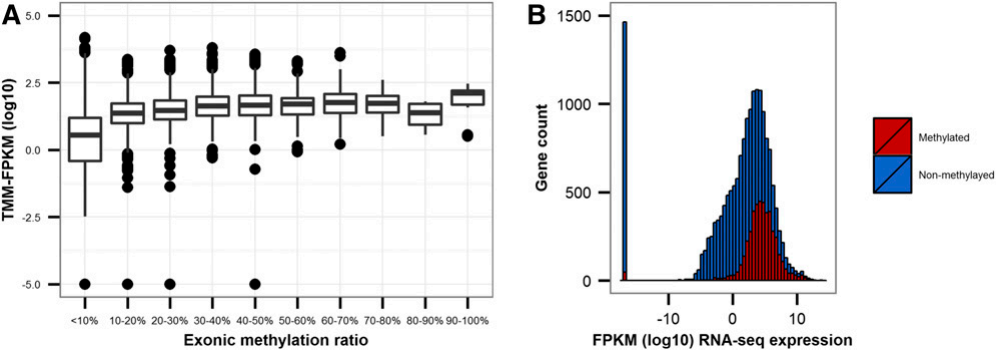
The relationship between methylation and gene expression in *H. armigera*. (A) Distribution of RNA sequencing (RNA-seq) TMM (Trimmed mean of *M*-values)-fragments per kilobase of exon per million fragments mapped (TMM-FPKM log_10_) expression stratified by exon methylation bins (10%). (B) Density plots for expression data for individual genes per methylated (red) or nonmethylated (blue) status, based on ± 10% exonic methylation.

The relationship between DNA methylation and gene expression was investigated using an RNA-seq data set from adult *H. armigera* collected from a nearby population in China (Jones *et al.* 2015). There was a largely positive, although nonlinear, relationship between intragenic methylation and expression (Spearman’s rank, ρ = 0.397, *P <* 0.0001) (Figure 3A). The median expression of methylated genes was significantly greater than that of those nonmethylated (± 10%) (Wilcoxon Signed-Rank test, *P <* 0.0001) (Figure 3B). It was also notable that of the 1462 genes not expressed (FPKM = 0), 81.0% had zero exonic methylation, a large increase from the percentage of genes that have no detectable exonic methylation throughout the genome (43.1%).

These results demonstrate that DNA methylation is tightly associated with stably expressed genes in *H. armigera* and that the function of methylation is likely to mirror that in other highly diverged insect orders (*e.g.*, Hymenoptera and Orthoptera) (Lyko *et al.* 2010; Flores *et al.* 2012; Hunt *et al.* 2013b; Wang *et al.* 2013). Given the observation that methylation is spatially correlated with histone modifications (Glastad *et al.* 2015), future studies exploring the regulation of gene expression in *H. armigera* (and other Lepidoptera) via DNA methylation should be investigated in the context of chromatin organization and the wider epigenetic landscape.

### Functional enrichment of methylated genes in H. armigera

A functional enrichment analysis of those genes exhibiting high exon methylation ratios (> 50%) showed that these genes are related to basic housekeeping roles such as ribosome structure, translation, and gene expression (Table S2). Conversely, genes lacking any mCpGs were enriched for specialized functions such as cell signaling (G-protein coupled receptors), detoxification, olfaction, and the insect cuticle (Table S3). This finding provides additional weight to the hypothesis that an important function of methylation in a diverse array of insects, including Lepidoptera, is the regulation of general cellular processes in ubiquitous, evolutionary conserved, and stably expressed genes (Elango *et al.* 2009; Hunt *et al.* 2010; Xiang *et al.* 2010; Wurm *et al.* 2011; Sarda *et al.* 2012).

### Validation of methylation in selected loci via targeted bisulfite sequencing

**Figure 4:**
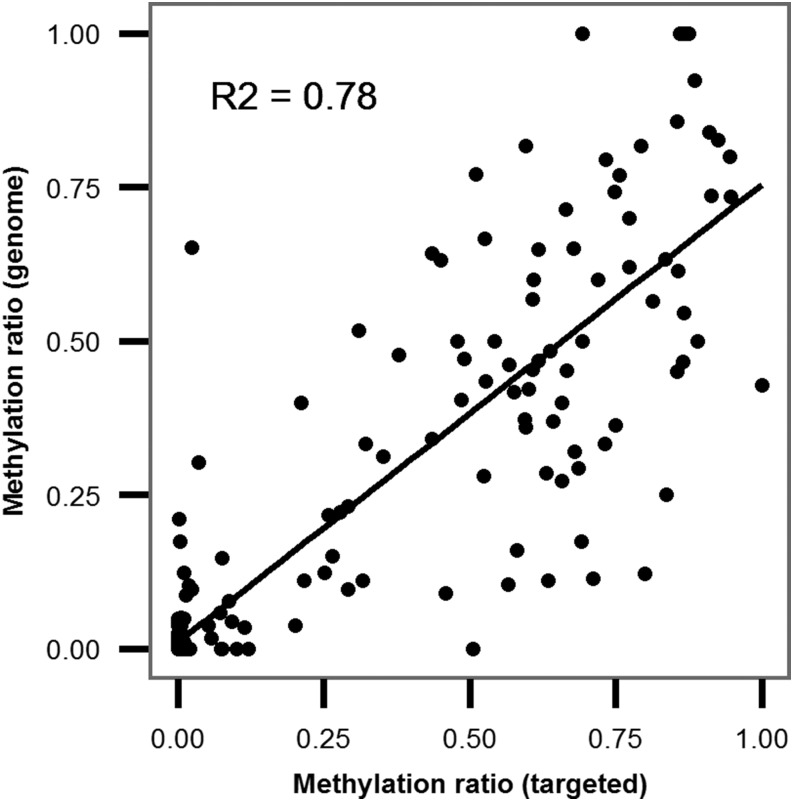
Correlation between methylation in selected loci analyzed by whole-genome and targeted sequencing.

To validate the whole-genome methylation data, primers were designed to targeted selected loci in 16 genes (see below for details). Excellent coverage was obtained with 305,680–507,593 reads per sample, an average CpG coverage ranging from 208× to 949× and a bisulfite conversion rate of > 99%. Following quality control, a total of 322 CpG sites were detected above the required threshold (> 10 reads per site) in either exonic or 5′-UTR regions (94 sites were detected in all 16 samples). A comparison of methylation levels from the whole genome *vs.* the average targeted bisulfite sequencing across all samples showed a strong positive relationship (*R*^2^ = 0.78 and *P* < 0.0001; Figure 4). The fact that the two methylation detection methods were strongly correlated, despite that fact they were performed on different adult *H. armigera* samples, suggests that the methylation status of most individual CpG sites is relatively stable across different individuals of this species.

### Methylation of selected genes associated with flight behavior

A whole-genome transcriptional analysis previously showed that the flight activity of *H. armigera* is associated with the differential expression of a suite of genes encompassing a range of biological functions, including fatty acid/ketone metabolism, flight muscle structure, and ATP synthesis/respiration (Jones *et al.* 2015). The mean exon methylation of these candidate genes (*n* = 191) is 0.040 (range 0–0.684) with an CpG O/E of 0.95; indicating similar, albeit slightly lower, levels compared to genome-wide methylation. A list of the 25 candidates with exon methylation ratios > 10% is provided in Table S4, with the highest levels present in the motor protein *dynein light chain roadblock-type 2* (HaOG207620), *NADH dehydrogenase* (HaOG208245), the lysine-specific demethylase *KDM4* (HaOG212852), and an ortholog of the *Drosophila* hypoxia-related gene *tnz CG4365* (HaOG210853). Selected loci from 16 candidates were chosen to validate the whole-genome analysis (Table S5).

To examine whether these genes also show signs of differential methylation in the context of flight behavior, a flight mill experiment was performed on *H. armigera* collected from northern Greece. Female moths showed continuous variation in flight performance with a mean total distance flown during a single night of 13,619 m. Flight mill data collected from multiple noctuid moth species (*H. armigera*, *Spodoptera frugiperda*, and *S. exempta*) indicate that insects that fly for longer distances, in general, engage in fewer flights (A. Pearson and C. M. Jones, unpublished data). Using this approach, we discriminated between long- (*N* = 8, mean distance = 21,586 m, and mean number of flights = 7.5) and short-distance fliers (*N* = 8, mean distance = 5246 m, and mean number of flights = 44.25) for comparison of methylation levels in the targeted gene set (Figure S3 in File S1).

**Figure 5:**
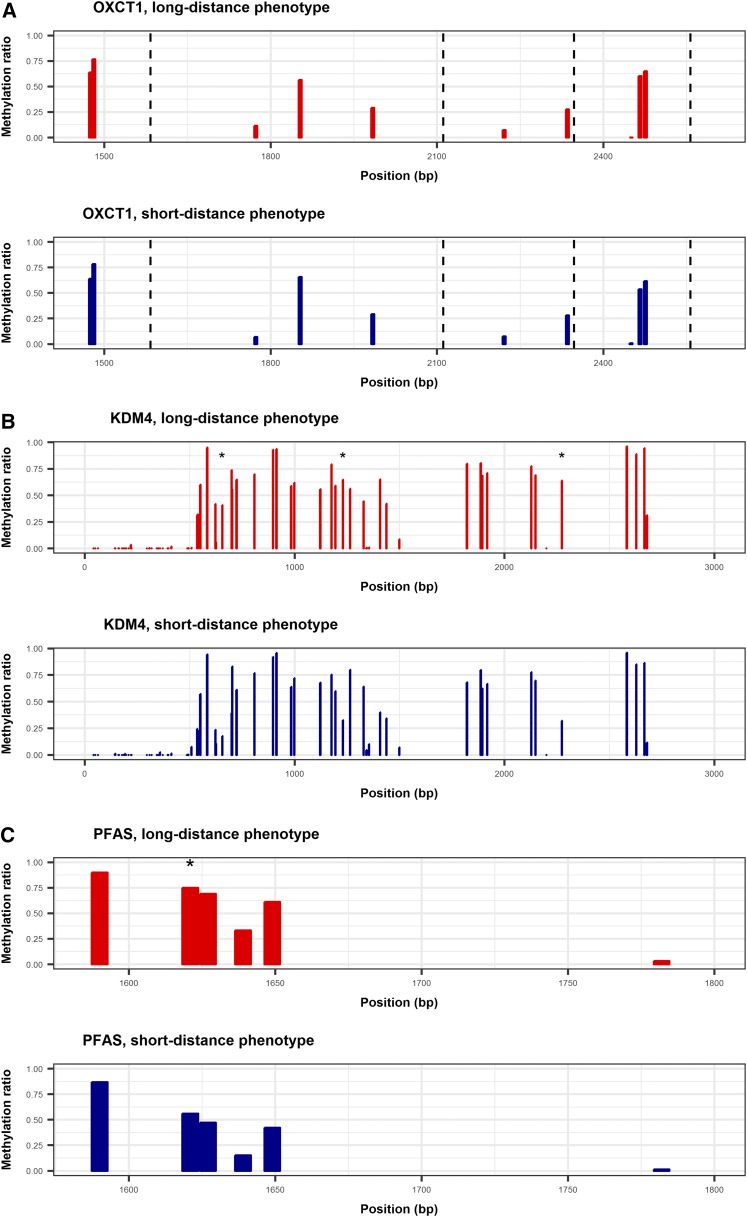
Methylation of selected loci in three genes as detected using targeted bisulfite sequencing. The bar graphs present the average methylation ratio at each CpG site detected for (A) succinyl-CoA:3-ketoacid coenzyme A transferase 1 (*OXCT*), (B) lysine-specific histone demethylase (*KDM4*), and (C) phosphoribosylformylglycinamidine synthase (*PFAS*). Methylation ratios were calculated as the average for each flight phenotype. The three CpG sites in *KDM4* and one site in *PFAS* that were significantly hypermethylated (*P* < 0.05) in long-distance flying insects are shown with * in the top panel. Dashed lines represent exon–exon boundaries for *OXCT*.

For the majority of loci, we observed few differences in the methylation levels between short- and long-distance fliers, with high concordance between the flight groups (*R*^2^ = 0.84 and *P* < 0.0001) (Figure S4 in File S1). For example, in the ketone metabolism gene succinyl-CoA:3-ketoacid coenzyme A transferase 1 (*OXCT*), the fractional methylation ratios per CpG site are almost identical across four exons (Figure 5A). This suggests that the transcriptional activity of many genes associated with flight performance in *H. armigera* is not influenced by DNA methylation (although in this preliminary study we have only looked at a comparatively small subset of previously identified candidate genes). However, there were two examples of genes (comprising a total of eight CpG sites) where methylation levels were significantly different between the flight phenotypes (Table 1).

**Table 1:** Hyper- and hypomethylated sites in selected loci when comparing short- and long-distance flight phenotypes

							Fractional Methylation (_M_CG/CG_ALL_)	
Gene Name	Description	Exon No.	Scaffold No.	Position	Meth. Diff.*^a^*	*P*-Value	Long-Distance	Short-Distance
HaOG212852	KDM4	1	480	8283	0.323	0.0007	0.648	0.325
HaOG212852	KDM4	1	480	7239	0.320	0.0312	0.639	0.319
HaOG212852	KDM4	1	480	8858	0.231	0.0474	0.408	0.177
HaOG206723	Phosphoribosylformylglycinamidine synthase-like	3	211	76644	0.190	0.0298	0.750	0.560
HaOG202339	Mobile element jockey-like	2	109	667671	0.005	0.0192	0.005	0.000
HaOG216422	Phosphorylated CTD-interacting factor 1-like	1	86	1009788	−0.024	0.0247	0.000	0.024
HaOG206745	Succinyl-CoA:3-ketoacid coenzyme A transferase 1	4	211	282391	−0.035	0.0467	0.902	0.937
HaOG202350	Phosphoribosyl pyrophosphate synthetase	1	11	223889	−0.044	0.0476	0.833	0.876

No., number; Meth. Diff., methylation difference; CTD, C-terminal domain; CoA, coenzyme A.

a Methylation differences between phenotypes determined by average methylation ratio across individual samples.

The top three hypermethylated sites in the long-distance fliers—with fractional methylation ratios 0.231–0.323 greater compared to short-distance fliers—were all present in *KDM4* (Table 1). In accordance with relatively high exonic methylation (∼50%) and a low CpG O/E value (0.58), a large percentage of CpG sites in *KDM4* were methylated (Figure 5B). *KDM4* encodes a demethylase that removes di- and trimethyl groups from lysines 9 and 36 in histone H3 (H3K9 and H3K36) (Klose *et al.* 2006), and therefore plays a role in reversing histone methylation, which itself is associated with transcriptional activity. The colocalization of DNA methylation and histone post-translational modifications (*e.g.*, H3K9me3 and H3K36me3) are strongly associated with stably expressed genes (Hunt *et al.* 2013b; Glastad *et al.* 2015). For example, Glastad *et al.* (2015) show that > 90% of methylated genes also feature H3K4me3 or H3K36me3. The consequences of hypermethylation in the *KDM4* gene itself in the context of an energetic activity such as flight, which requires a strong transcriptional response, is unknown. It has been shown that the loss of *KDM4* in *Drosophila* impedes the transcriptional activation of ecdysone signaling (Tsurumi *et al.* 2013), a pathway with increasingly recognized importance in adult insect behavior (Schwedes and Carney 2012).

A functional enrichment analysis has previously shown that genes associated with the inosine monophosphate biosynthesis pathway and purine/ATP metabolism are enriched in overexpressed genes associated with increased flight activity (Jones *et al.* 2015). However, genes with these GO terms are not highly methylated (mean exonic methylation = 0.035) except for *PFAS*, an enzyme that encodes phosphoribosylformylglycinamidine synthase. This gene contained the only other strongly differentially methylated site between the flight phenotypes (Figure 5C and Table 1). This enzyme catalyzes part of the pathway involved in ionosine purine biosynthesis and ATP turnover, but whether the expression of this pathway induced by the demands of a highly energetic activity such as migratory flight requires mediation via a hypermethylated site requires further investigation.

While DNA methylation in the exonic regions of insect genomes is associated with transcription, this methylation largely occurs in genes with basic regulatory functions and generally not in those genes that are differentially expressed between phenotypes (Hunt *et al.* 2013a; Libbrecht *et al.* 2016; Sarda *et al.* 2012). Indeed, the function of DNA methylation in the context of expression in insects is still largely unknown and is likely to require further study using all components of the epigenome (Glastad *et al.* 2016). In this context, it is unlikely that differential methylation will contribute largely to the contrasting flight capacities exhibited by *H. armigera* in this study. Nevertheless, the differentially methylated sites described above do represent viable targets to determine the functional significance of methylation on expression and/or flight behavior. At single-base resolution, the induction of methylation *in vivo* via the CRISPR/Cas9-based system (McDonald *et al.* 2016) represents a promising future application to determine the role of differentially methylated sites in insects. At the genome-wide scale, chemical disruption of methylation via a demethylating agent has been shown to lead to subtle changes in sex allocation in the parasitic wasp *Nasonia vitripennis* (Cook *et al.* 2015). Migration is a complex syndrome consisting of a combination of several morphological, behavioral, and physiological traits (Liedvogel *et al.* 2011; Chapman *et al.* 2015). Therefore, it seems plausible that the disruption of DNA methylation in migratory insects containing a functional methylation system, including *H. armigera*, could also result in subtle but significant changes in one of the many biochemical pathways that contribute to this behavior. The knockdown of methyltransferases via CRISPR or RNA interference [*e.g.*, Flores *et al.* (2012)] also represents a potential experimental tool.

### Conclusions

The description of the single-base-resolution methylome of *H. armigera* presented here provides an insight into genome-wide DNA methylation in a noctuid moth. Our findings reveal that, as reported for other insects, methylation is sparse in this species, with close to ∼1% of CpG sites identified as methylated, in sharp contrast to the 60–90% methylation levels observed in mammals. Methylation in *H. armigera* is predominantly exonic and significantly enriched in genes involved in basal cellular housekeeping roles. The degree of genic methylation in this species is positively correlated with gene expression, although the relationship is not linear, with methylated genes exhibiting higher median expression levels than nonmethylated genes, consistent with the results of other insect species. Recent studies have provided some initial evidence of a relationship between methylation and life history divergence associated with long-distance migration (Wang *et al.* 2014; Baerwald *et al.* 2016). Our preliminary exploration of the role of this epigenetic mark in the regulation of the expression of candidate genes associated with this trait in *H. armigera* suggests the that transcription of only a minor subset of genes may be influenced by methylation. However, these genes represent promising candidates for further characterization in the context of methylation and other epigenetic marks, such as histone modifications. Finally, we envisage that the *H. armigera* methylome will be a valuable resource for further research into the epigenetic control of adaptive traits in this important insect pest [*e.g.*, resistance to Bt toxins and insecticides (Downes *et al.* 2016)], especially now that the full genome is available (Pearce *et al.* 2017).

## Supplementary Material

Supplemental material is available online at www.g3journal.org/lookup/suppl/doi:10.1534/g3.117.1112/-/DC1.
