# Supplementary material for: Genome-Wide Characterization of DNA Methylation in an Invasive Lepidopteran Pest, the Cotton Bollworm Helicoverpa armigera
Source: G3 (Bethesda). 2018 Jan 2;8(3):779–87. doi: 10.1534/g3.117.1112 (PMC5844299; doi:10.1534/g3.117.1112)
Supplement: Supplementary file 7 [file 779FileS1.docx]

**Supplemental Figures**

**Genome-wide characterisation of DNA methylation in an invasive Lepidopteran pest, the cotton bollworm *Helicoverpa armigera***

Christopher M. Jones^*†^, Ka S. Lim^‡^, Jason W. Chapman**^§^** & Chris Bass**^**^**

**^*^Vector Biology, Liverpool School of Tropical Medicine, Pembroke Place, Liverpool, L3 5QA, UK**

**^†^Biointeractions and Crop Protection, Rothamsted Research, Harpenden, Hertfordshire, AL5 2JQ, UK**

^‡^**Computational and Analytical Sciences, Rothamsted Research, Harpenden, Hertfordshire, AL5 2JQ, UK**

^‡^**Centre for Ecology and Conservation, University of Exeter, Penryn, Cornwall TR10 9EZ, UK**

**^**^College of Life and Environmental Sciences, University of Exeter, Penryn, Cornwall TR10 9EZ, UK**

**Corresponding author:** Christopher Jones, Vector Biology, Liverpool School of Tropical Medicine, Pembroke Place, Liverpool, L3 5QA, UK

[chris.jones@lstmed.ac.uk](mailto:chris.jones@lstmed.ac.uk)


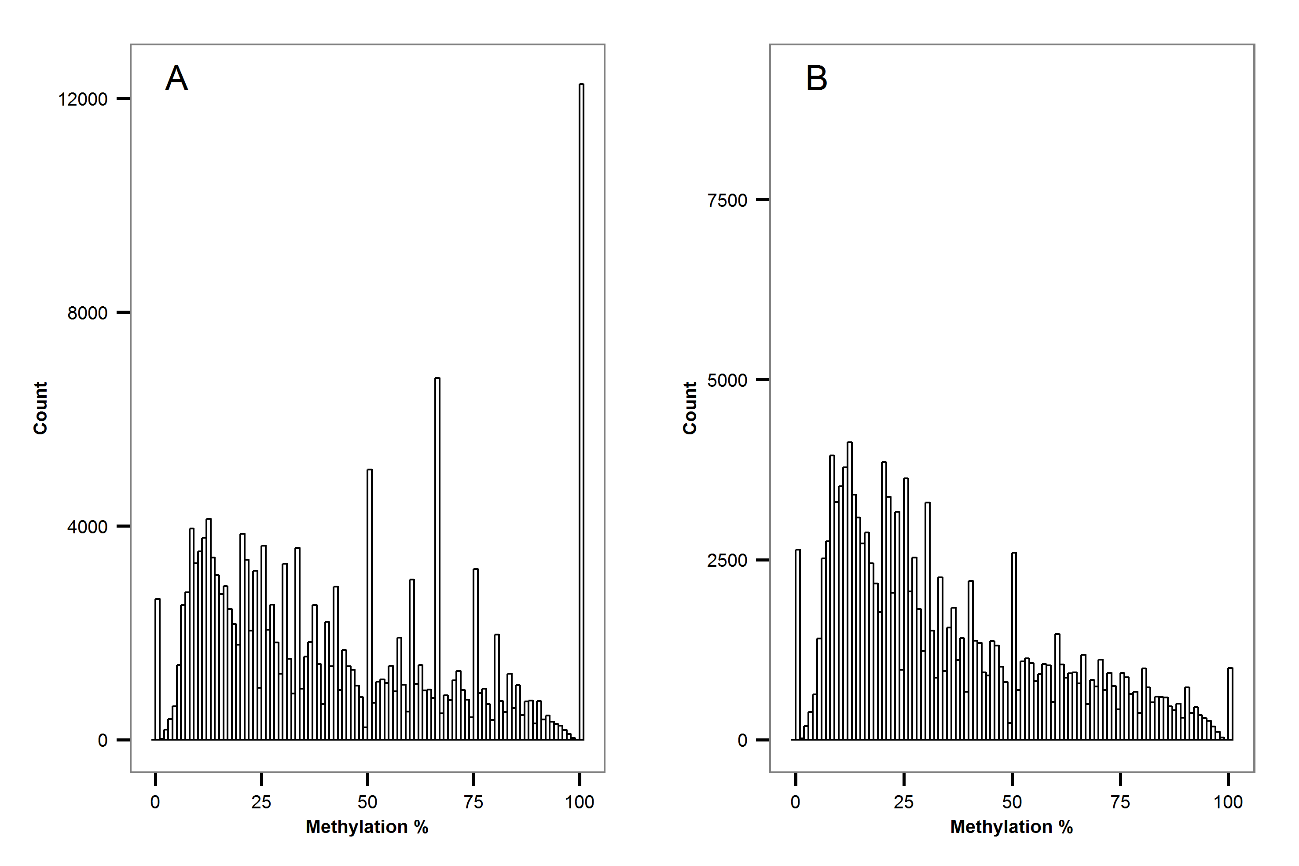


**Figure S1.** Distribution of methylated CpG sites according to the percentage of methylated reads for A) at least 2 reads per site and B) at least 10 reads per site.

**
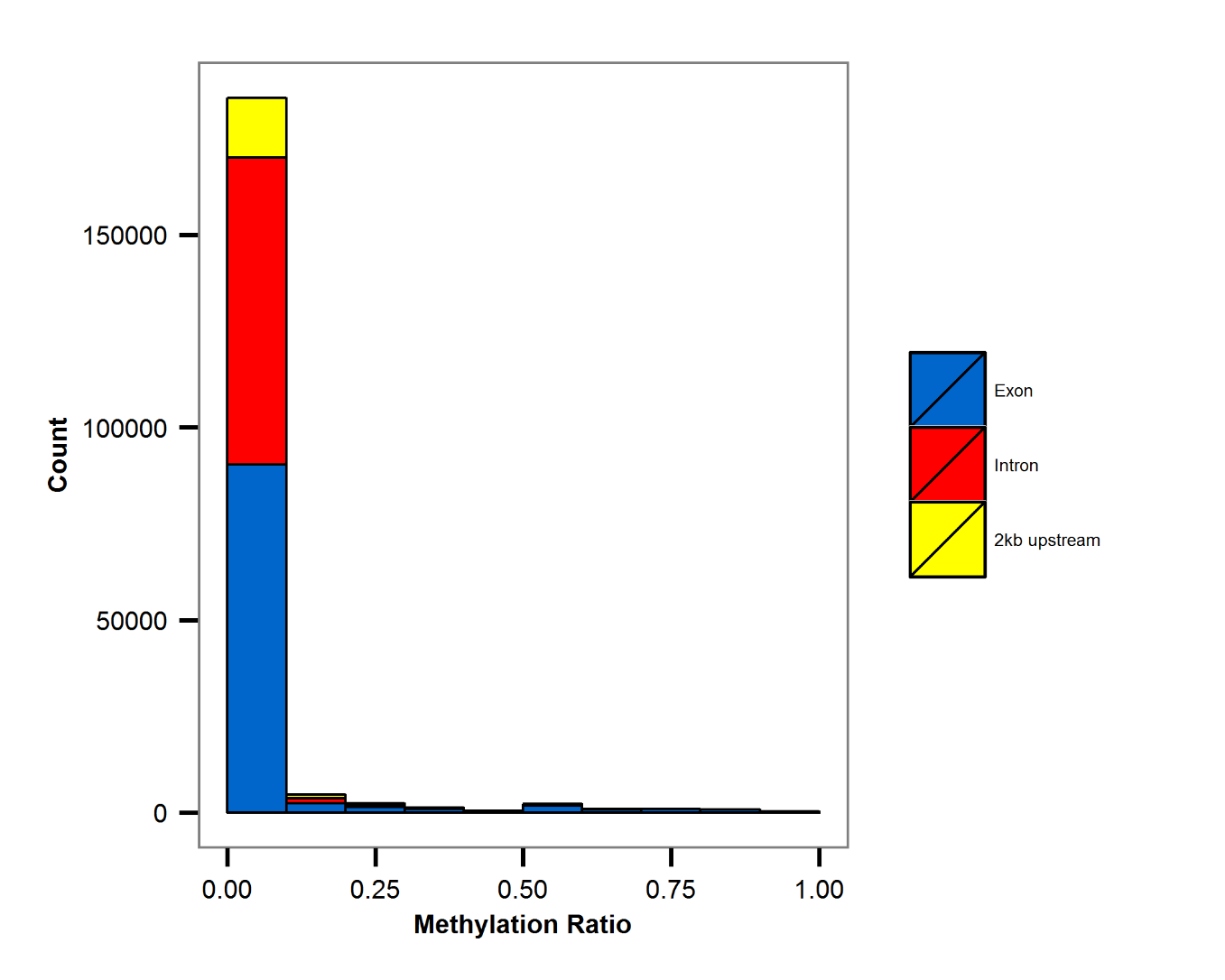
Figure S2.** DNA methylation in *H. armigera* per genomic function. Data presented for exon, intron and 2kb upstream of the transcription start site.


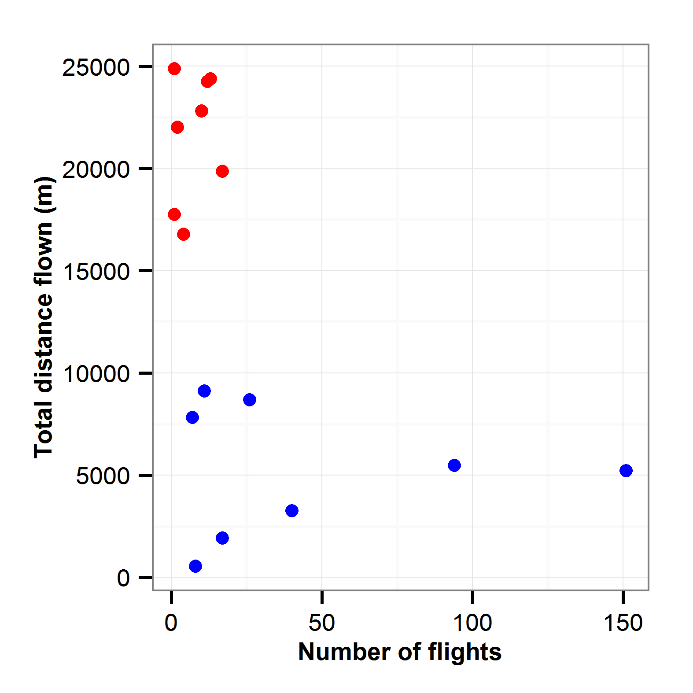


**Figure S3.** Phenotyping of adult *H. armigera* moth flight behaviour.

Scatter plot of the total distance flown versus the number of flights undertaken by individual moths. Insects selected for targeted bisulfite sequencing group into two distinct clusters; long distance (red) or short-distance (blue) fliers.


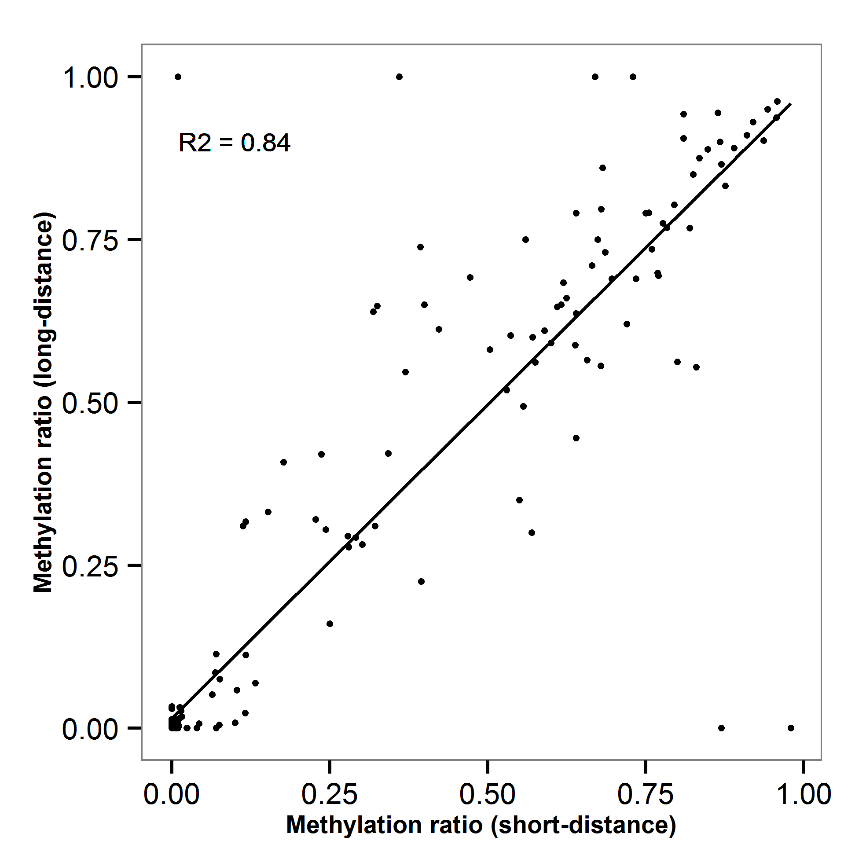


**Figure S4.** Correlation between the methylation ratio in individual CpG sites averaged across samples belonging to the short- and long-distance phenotypes.
